# Evaluating the Presence of Empathic Communication in ChatGPT-Produced Clinical Notes Using Established Communication Frameworks

**DOI:** 10.7759/cureus.102750

**Published:** 2026-01-31

**Authors:** Sydney Bowden, Keyline Moreno, Frederick Million, Nicholas Azinge

**Affiliations:** 1 Internal Medicine, Howard University College of Medicine, Washington, D.C., USA; 2 Internal Medicine, Howard University Hospital, Washington, D.C., USA

**Keywords:** artificial intelligence, chatgpt, clinical documentation, empathic communication, ethical considerations, patient centered care

## Abstract

Background: Empathy is a core component of effective physician-patient communication and is associated with improved clinical relationships and patient experience. As generative artificial intelligence (AI) models such as ChatGPT (OpenAI, San Francisco, California, United States) are increasingly explored for clinical documentation support, it is important to understand whether these systems can produce language that reflects empathic communication.

Objective: This study evaluated empathic communication in ChatGPT-generated clinical notes through two distinct approaches: (i) quantitative measurement of linguistic markers using established communication frameworks, and (ii) qualitative characterization of empathic styles and patterns.

Methods: A cross-sectional simulation study was conducted using ChatGPT (large language model, web-based interface, December 2025 version 5.1). Twenty standardized pediatric cases were created across psychiatry and gastroenterology contexts. For each case, two Subjective, Objective, Assessment, and Plan (SOAP) format clinical notes were generated under different prompting conditions: a neutral clinical tone and an explicitly empathetic clinical tone. Notes were evaluated using the Consultation and Relational Empathy (CARE) Measure and the Empathic Communication Coding System (ECCS).

Results: Forty clinical notes were analyzed by two independent blinded raters. Notes generated under empathetic prompting demonstrated higher mean CARE scores compared with neutral notes (3.8 vs. 2.6 on a five-point scale), with differences found to be statistically significant (two-tailed independent samples *t*-test, *p* < 0.001). Empathetic-tone notes also contained a greater frequency of empathic statements as measured by the ECCS. Empathic language primarily reflected generic supportive phrasing, cognitive acknowledgment of patient concerns, and action-oriented reassurance, while context-specific emotional nuance remained limited across both prompting conditions.

Conclusions: ChatGPT can generate clinical documentation containing measurable expressions of empathy when explicitly prompted; however, this empathy remains largely formulaic and dependent on prompt design. These findings highlight both the potential utility and limitations of generative AI tools in producing patient-centered clinical documentation.

## Introduction

Empathy, defined as the ability to understand and appropriately respond to the feelings and experiences of another, is a cornerstone of effective medical practice. Higher levels of physician empathy have been associated with improved diagnostic accuracy, greater patient adherence, and increased patient satisfaction [1]. Additional work in primary care and chronic disease management has demonstrated that clinician empathy is associated with improved patient trust, engagement, and clinical outcomes, reinforcing empathy as a measurable and clinically meaningful component of care [2-4]. Beyond its interpersonal value, empathy is increasingly recognized as a professional and ethical dimension of clinical care that is reflected not only in patient interactions but also in clinical documentation.

As healthcare systems adopt digital tools to address growing documentation demands, artificial intelligence (AI)-based systems are becoming increasingly integrated into clinical workflows. Large language models such as ChatGPT (OpenAI, San Francisco, California, United States) have demonstrated proficiency in generating clinical notes, patient education materials, and decision support text. While prior research has largely focused on the accuracy, safety, and reasoning capabilities of these systems, far less attention has been given to their capacity to convey empathic communication through written clinical documentation.

Emerging evidence suggests that ChatGPT can produce responses perceived as empathetic in simulated patient-facing scenarios, in some cases scoring comparably to human clinicians on empathy assessments [5]. However, these evaluations have primarily examined conversational exchanges rather than structured clinical notes. Clinical documentation serves a distinct function. It communicates clinical reasoning, reflects professional tone, and signals attentiveness to patient concerns within a standardized format. Whether AI-generated notes can meaningfully reflect empathic communication within these constraints remains insufficiently explored.

This study aimed to evaluate empathic communication in ChatGPT-generated clinical notes using a mixed-methods approach. Quantitatively, we sought to measure the frequency and magnitude of linguistic empathy using the Consultation and Relational Empathy (CARE) Measure and the Empathic Communication Coding System (ECCS). Qualitatively, we aimed to characterize the dominant styles and thematic patterns of AI-generated empathy. Focusing on pediatric psychiatry and gastroenterology contexts, settings in which emotional sensitivity and patient-centered documentation are particularly important, we assessed whether empathic language could be systematically measured, differentiated by prompting conditions, and thematically characterized. Clarifying the capabilities and limitations of AI-generated empathy in documentation is essential as these tools continue to shape clinical practice and medical education.

## Materials and methods

Study design

This was a fully AI-based cross-sectional study conducted using ChatGPT (large language model (LLM), web-based interface, December 2025 version 5.1). All data was generated within the ChatGPT interface. ChatGPT was selected because it represents one of the most widely used and clinically evaluated LLM platforms for medical documentation research, making it a relevant system for assessing empathic language generation in contemporary clinical contexts. No real patient information or protected health data were entered into the model; all cases were fully synthetic and generated by the authors. No human participants, patient data, or protected health information were used. Clinical notes in the United States are routinely accessible to patients through electronic health record portals under OpenNotes (Beth Israel Deaconess Medical Center, Boston, Massachusetts, United States) policies, and the generated notes in this study were evaluated in this context. The study was exempt from institutional review board review because it involved only synthetically generated data. Standardized case prompts included age, presenting symptoms, and a limited psychosocial context but did not explicitly specify patient race, ethnicity, socioeconomic status, or underserved status. Prompts were intentionally designed to minimize demographic cues in order to isolate linguistic expressions of empathy independent of explicit identity attributes.

Case generation

A total of 10 simulated pediatric psychiatry cases and 10 pediatric gastroenterology cases were created. Each case included brief background details such as patient age, presenting symptoms, and limited psychosocial context. Prompts did not explicitly specify patient race, ethnicity, socioeconomic status, or underserved status. An example prompt was:

“Write a Subjective, Objective, Assessment, and Plan (SOAP) note for a 15-year-old with Crohn’s disease who reports fatigue, anxiety, and difficulty managing school stress. Include patient concerns, clinician reflections, and a management plan.”

Prompting conditions

For each case, ChatGPT generated two versions of the clinical note under different prompting conditions: a neutral clinical tone and an explicitly empathetic clinical tone. This resulted in a total of 40 clinical notes, including 20 neutral tone notes and 20 empathetic tone notes. The base clinical prompt was identical across all cases. The only variation introduced was a brief instruction specifying either a neutral clinical tone or an empathetic tone, ensuring that observed differences were attributable to tone rather than content variation. To minimize potential memory or context carryover effects, each prompt was entered into a new, independent ChatGPT session, and no prior conversational context was retained between cases.

Empathy evaluation

Each clinical note was evaluated using the CARE Measure and the ECCS to assess observable linguistic markers of empathic communication, not affective intent or emotional understanding [6,7]. Two independent raters scored all notes, and mean scores were calculated across raters and prompting conditions. Raters were blinded to prompting conditions during scoring to minimize bias.

The CARE Measure is a validated patient-reported outcome instrument developed by Mercer et al. to assess clinician empathy across domains such as understanding patient concerns, showing compassion, and fostering a supportive therapeutic relationship [6]. Although the CARE Measure is publicly available for clinical use within the United Kingdom, it is a copyrighted instrument requiring permission for research use outside the UK. Written permission for use of the CARE Measure in this study was obtained directly from the copyright holder, Professor Stewart Mercer, prior to initiation of the study. Scores are reported on a five-point Likert scale, with higher scores indicating greater perceived empathy.

The ECCS, developed by Bylund and Makoul [7] and informed by foundational work on empathic communication by Hall et al. [8], is a previously published qualitative coding framework used to identify and categorize empathic clinician communication. In accordance with the tool’s stated usage requirements, permission to use the ECCS for research purposes was obtained from the instrument’s developer, Dr. Carma Bylund, prior to analysis. ECCS coding was applied solely for research purposes to identify and quantify discrete empathic statements within each AI-generated note.

Data analysis

Quantitative comparisons between prompting conditions were performed descriptively using means, standard deviations, and t tests. Qualitative thematic analysis was conducted to identify recurring patterns of empathic expression [9].

## Results

Representative excerpts

Representative excerpts from ChatGPT-generated SOAP notes for the same standardized pediatric gastroenterology case under neutral and empathetic prompting conditions are given below.

Neutral Clinical Tone (Excerpt)

“Patient reports fatigue and abdominal discomfort consistent with Crohn’s disease. Symptoms have interfered with school attendance. Plan includes continuation of current biologic therapy and referral to gastroenterology for follow-up.”

Empathetic Clinical Tone (Excerpt)

“Patient expresses significant fatigue and frustration related to ongoing Crohn’s disease symptoms, noting difficulty keeping up with school demands. These concerns were acknowledged, and reassurance was provided that symptom burden is recognized and will be addressed through continued medical management and supportive resources.”

These excerpts demonstrate that empathic prompting increased acknowledgment of patient experience and psychosocial context while preserving clinical assessment and management decisions.

CARE and ECCS scoring

Quantitative empathy measures differed significantly between prompting conditions, with higher CARE scores and a greater number of empathic statements observed in empathetic-tone notes (Table 1).

**Table 1:** Comparison of empathy scores between prompting conditions CARE: Consultation and Relational Empathy; ECCS: Empathic Communication Coding System

Prompting Condition	CARE Score, mean±SD	ECCS (Empathic Statements per Note), mean
Neutral tone	2.6±0.5	1.3
Empathetic tone	3.8±0.4	3.9

Clinical notes generated under a neutral tone demonstrated a mean CARE score of 2.6 (SD = 0.5) and an average of 1.3 empathic statements per note as measured by the ECCS. In contrast, notes generated under an empathetic tone demonstrated a higher mean CARE score of 3.8 (SD = 0.4) and an average of 3.9 empathic statements per note. Differences between prompting conditions were statistically significant (two-tailed independent samples t-test: t = 7.84, df = 38, p < 0.001).

CARE Measure domain scores and ECCS category frequencies are given in Tables 2, 3, respectively, according to the prompting condition.

**Table 2:** CARE Measure domain scores by prompting condition CARE: Consultation and Relational Empathy

CARE Domain	Neutral Tone, mean ± SD	Empathetic Tone, mean ± SD
Making patient feel at ease	2.5 ± 0.6	3.9 ± 0.4
Letting patient tell story	2.6 ± 0.5	3.7 ± 0.4
Showing care and compassion	2.4 ± 0.6	4.0 ± 0.3
Understanding patient concerns	2.7 ± 0.5	3.8 ± 0.4
Helping patient take control	2.8 ± 0.4	3.6 ± 0.5

**Table 3:** ECCS category frequency per clinical note ECCS: Empathic Communication Coding System

ECCS Category	Neutral Tone	Empathetic Tone
Emotional acknowledgment	0.4	1.5
Reflective statements	0.5	1.2
Validation of concerns	0.3	0.9
Supportive reassurance	0.1	0.3
Total empathic statements	1.3	3.9

Qualitative thematic analysis identified three dominant empathy styles: generic supportive statements, cognitive empathy, and action-oriented empathy. Across both prompting conditions, the incorporation of individualized psychosocial context beyond information explicitly provided in the prompt was limited.

## Discussion

This study demonstrates that ChatGPT can produce measurable linguistic expressions of empathic communication in clinical documentation when explicitly instructed to do so, rather than demonstrating empathy as an affective or experiential capacity. The observed differences between neutral and empathetic prompting conditions indicate that empathic expression in AI-generated clinical notes is prompt-dependent rather than intrinsic. A neutral-tone AI prompt was selected as the comparator condition to isolate the effect of empathic prompting within the same model and documentation structure, thereby minimizing confounding related to inter-author variability. Recent reviews of LLMs and empathy suggest these systems can approximate aspects of cognitive empathy in text generation, while evaluation approaches remain heterogeneous and context-dependent [10]. These findings suggest that empathic language produced by LLMs reflects structured linguistic patterns rather than spontaneous or internally motivated emotional understanding.

Although ChatGPT reproduced recognizable empathic structures, including acknowledgment of concerns and supportive phrasing, the generated notes consistently lacked contextual depth. Empathy in this setting is therefore best conceptualized as linguistic imitation rather than affective understanding. While the model can replicate the outward form of empathic communication, it does not possess awareness, intention, or emotional experience, which are central to human empathy.

Prior research has shown that ChatGPT responses in public and simulated conversational settings may be perceived as more empathetic than physician responses [1]. Our findings extend this literature by demonstrating that empathic language can also be generated within structured clinical documentation. This distinction is important, as clinical notes serve not only as a record of care but also as a reflection of professional tone and patient-centeredness within standardized formats.

From a practical perspective, AI-generated empathic language may have value as a supportive tool in drafting patient-centered documentation or as an educational resource for teaching empathic communication. In parallel, emerging work evaluating LLM-generated clinical notes highlights both potential documentation gains and persistent quality risks, reinforcing the need for validated assessment frameworks and human oversight [11]. However, the use of simulated empathy in clinical records raises important ethical considerations. Language that appears empathic may influence patient perceptions or institutional evaluations of care quality, even though the sentiment itself originates from an algorithm rather than a clinician.

Several limitations should be acknowledged. This study evaluated outputs from a single AI model, and findings may not generalize to other systems or future model iterations. The rapid pace of LLM development represents an additional important limitation. Model architectures, training data, safety constraints, and alignment strategies are updated frequently, and empathic language generation capabilities may change substantially across successive model versions. As a result, the findings of the present study reflect the behavior of a specific ChatGPT deployment at a defined point in time and may not generalize to future model iterations or alternative platforms. Future research should therefore incorporate longitudinal evaluations across model versions and comparative analyses across systems to assess the stability, evolution, and generalizability of AI-generated empathic language over time. In addition, empathy assessment tools such as CARE and ECCS were developed for human interactions and may not fully capture the nuances of AI-generated text. Subjective interpretation of empathic language also introduces variability, despite the use of multiple raters. Importantly, while ChatGPT can imitate the cognitive and linguistic components of empathy, it lacks the affective dimension that arises from human relational context [12]. 

Additionally, the absence of a human clinician-authored comparator limits direct conclusions about how AI-generated empathic language compares with clinician-written documentation. Prior studies have demonstrated that perceived clinician empathy is associated with improved adherence, trust, and clinical outcomes, reinforcing the importance of empathic communication even within structured documentation formats [2-4]. This distinction is ethically significant, as documentation that appears empathic may convey a sense of understanding that does not reflect human engagement. Some scholars argue that simulated empathy may still have pragmatic value if it improves clarity, tone, and patient-centeredness when used transparently as an assistive tool [4]. Conversely, others caution that AI-generated empathy may blur moral accountability and risk misleading clinicians or patients regarding the authenticity of compassion expressed in clinical documentation [12]. 

The ethical acceptability of simulated empathy in clinical documentation likely depends on intent, context, and disclosure. When used deliberately and transparently to support clinician communication, AI-generated empathic language may enhance patient-centered documentation. When used uncritically or without acknowledgment, however, it may undermine trust in the authenticity of clinical communication. These findings highlight the need for clear guidelines governing the ethical use of AI-assisted documentation.

Beyond concerns of authenticity, the integration of AI-assisted empathic language into clinical documentation raises important questions regarding bias, equity, and standardization. Emerging evidence suggests that LLMs may reproduce societal and demographic biases present in their training data, potentially leading to differential expressions of empathy based on patient characteristics such as race, gender, or socioeconomic context [13,14]. In clinical settings, such disparities could inadvertently reinforce existing inequities in patient-provider communication. Additionally, the routinization of AI-generated empathic phrasing risks homogenizing clinical narratives, potentially diminishing individualized reflection and clinical reasoning if over-relied upon [15]. These concerns highlight the need for rigorous evaluation frameworks, transparency in AI deployment, and clear delineation of AI’s role as a supportive, rather than substitutive, tool in clinician communication and documentation [16].

Although medical education outcomes were not directly assessed in this study, the findings may have relevant implications for medical education, particularly in the context of teaching empathic communication and clinical documentation skills. As LLMs such as ChatGPT are increasingly explored as educational tools, AI-generated clinical notes may serve as illustrative examples to help trainees recognize structured elements of empathic language within standardized documentation formats. Exposure to AI-generated notes that explicitly incorporate empathic phrasing may support reflective learning, prompting students to critically evaluate how empathy is conveyed linguistically and how it can be integrated authentically into their own clinical writing. However, reliance on AI-generated empathy also carries pedagogical risks. The formulaic nature of the empathic language observed in this study underscores the importance of emphasizing human relational context, emotional attunement, and lived patient experience in medical training. Educators should frame AI-assisted documentation as a supplementary tool rather than a substitute for experiential learning, mentorship, and direct patient interaction in developing genuine empathic competence.

Importantly, this study did not explicitly examine how AI-generated empathic language may vary across patient race, ethnicity, or underserved contexts. Prior scholarship has demonstrated that algorithmic systems can perpetuate or amplify existing healthcare disparities when trained on biased data or deployed without equity-informed design. The absence of demographic specification in our prompts represents a limitation, as it precludes assessment of whether empathic language would differ across marginalized or minority patient scenarios.

Future research should incorporate diverse demographic, socioeconomic, and underserved patient contexts into prompt design to evaluate whether AI-generated empathic language remains consistent or exhibits bias or stereotyping across populations. Such an investigation is critical to ensuring that the adoption of generative AI in clinical documentation does not inadvertently reinforce or exacerbate disparities in perceived compassion, patient trust, or quality of care. Additionally, comparative studies examining AI-generated empathic language alongside clinician-authored documentation are needed to assess differences in authenticity, contextual sensitivity, and potential demographic bias, as well as to inform the development of hybrid human-AI documentation models that balance efficiency with relational integrity.

## Conclusions

ChatGPT can generate clinical documentation that contains measurable expressions of empathic language when explicitly prompted to do so, but this should not be interpreted as evidence of authentic or affective empathy. Rather, this empathy is conditional and synthetically produced, reflecting patterned linguistic expression rather than genuine emotional understanding. While AI-generated empathic language may support patient-centered tone and clarity in clinical notes, it does not replace the relational and affective components of human empathy. Recognizing these limitations is essential as generative AI tools become increasingly integrated into clinical documentation, medical education, and healthcare workflows.

As generative AI continues to shape clinical practice and training, understanding how empathic language can be induced, measured, and ethically interpreted will be increasingly important. While AI-generated empathy cannot replace human relational engagement, structured use of such tools may support patient-centered tone, educational reflection, and documentation quality when deployed transparently and under appropriate human oversight. Continued evaluation across evolving models, clinical contexts, and comparative human benchmarks will be critical to guide responsible integration of AI-assisted documentation into healthcare practice.
